# Neutron scattering studies of porous material

**DOI:** 10.1063/4.0001060

**Published:** 2025-10-27

**Authors:** CHENG LI

**Affiliations:** ORNL, Oak Ridge, TN, USA

## Abstract

Advances in porous materials—such as metal-organic frameworks (MOFs) and zeolites—offer promising pathways for developing next- generation separation systems, which account for 10–15% of global energy consumption, with improved selectivity, capacity, and throughput under ambient conditions. Realizing these benefits, however, requires experimental systems capable of mimicking realistic, dynamic, multicomponent gas environments.

Neutrons are uniquely suited for this area of research due to their sensitivity to light elements (e.g., hydrogen, carbon, and oxygen), ability to penetrate complex sample environments, and capacity to provide atomic-scale information on adsorption sites, host–guest interactions, and diffusion mechanisms. These insights are critical for unraveling the structure–property relationships that govern separation performance.

Here in, I will report the most recent neutron scattering studies of porous material conducted at the POWGEN instrument. I will discuss 1) the stepwise H_2_/D_2_ adsorption isotherm in M(ta)_2_ (M = Ni and Zn) systems and its structural origin using combined elastic and inelastic neutrons scattering. 2) ethylbenzene isomers separation using a NiFA framework materials and 3) He/H_2_ separation in the KAUST-7 material.

